# Cognitive Impairment in Inpatients with Prurigo Nodularis and Psychiatric Comorbidities

**DOI:** 10.3390/ijerph18126265

**Published:** 2021-06-09

**Authors:** Giuseppe Lanza, Filomena Irene Ilaria Cosentino, Raffaele Ferri, Bartolo Lanuzza, Maddalena Siragusa, Mariangela Tripodi, Carmelo Schepis

**Affiliations:** 1 Department of Surgery and Medical-Surgical Specialties, University of Catania, Via Santa Sofia 78, 95123 Catania, Italy; 2 Oasi Research Institute-IRCCS, Via Conte Ruggero 73, 94018 Troina, Italy; fcosentino@oasi.en.it (F.I.I.C.); rferri@oasi.en.it (R.F.); blanuzza@oasi.en.it (B.L.); msiragusa@oasi.en.it (M.S.); mtripodi@oasi.en.it (M.T.); cschepis@oasi.en.it (C.S.)

**Keywords:** itch, pruritus, chronic scratch lesions, psychiatric comorbidity, psychoactive drugs

## Abstract

Background: *Prurigo nodularis* (PN) is a chronic refractory itchy dermatosis. Although psychiatric comorbidity is known, research in cognitive impairment is lacking. We evaluated the occurrence and types of cognitive impairment in a series of inpatients with PN. Methods: This was a retrospective chart review of all the patients with PN admitted to a referral neurological institute from September 2018 to March 2021. Any neurological and psychiatric disorder, along with neuroactive drugs taken, were concomitantly assessed. Results: A total of 16 patients with PN (median age: 70 years, two males) were selected from a total of 1806 hospital admissions. Most of them had a neurodegenerative cognitive disorder, from mild cognitive impairment (8) to Alzheimer’s disease (1), followed by mixed disorder (degenerative and vascular) in six and vascular dementia in one. Comorbid psychiatric diseases (anxiety and depression) were more common than either individual condition, followed by bipolar disorder, whereas two patients did not show psychiatric manifestations. Most patients were on combined treatment with benzodiazepines and antidepressants. Conclusion: Cognitive impairment can be observed in PN. In addition to screening for psychiatric comorbidity and initiating appropriate treatment or referral, clinicians may also consider the presence of cognitive impairment in PN of both degenerative and vascular origin.

## 1. Introduction

*Prurigo nodularis* (PN) is a chronic difficult-to-treat itchy dermatosis of unknown etiology [1]. Typically, PN presents single-to-multiple symmetrically distributed, hyperkeratotic, and intensively itching papules and nodules, evolving in the context of different dermatological, systemic, psychiatric, or neurological conditions. Permanent scratching is a major trigger of PN, although its exact pathophysiology remains unclear [2]. Currently, therapy consists of topical steroids, capsaicin, calcineurin inhibitors, ultraviolet therapy, systemic administration of gabapentinoids, μ-opioid receptor antagonists, antidepressants, or immunosuppressants. Novel treatments, such as inhibitors of neurokinin-1 and interleukin-31 (IL-31) receptors, are currently being tested [3].

PN is supposed to be influenced by and associated with psychological factors, but research in this area is still scarce. Moreover, the relationships between dermatological and neuropsychiatric features are common and complex. Chronic nature of the dermatoses, disfigurement, impaired quality of life (QoL), and social stigma, indeed, are often associated with skin disorders and likely to be responsible for this association or, at least, involved in their pathophysiology. The association is bidirectional, and accurate screening and adequate management of psychiatric comorbidity, as well as treatment of skin conditions, aimed at the improvement of QoL may positively influence each other [4].

Some studies focusing on psychological symptoms and traits found that anxiety and depressive symptoms, as well as some traits such as alexithymia, are common in PN [5,6]. A clinical study showed that dermatological outpatients with common mental disorders also included subjects with PN [7]. Another registry-based audit confirmed that patients with PN are frequently diagnosed with anxiety and depression and are often prescribed psychotropic drugs [8]. A more recent study [4] aimed to assess the prevalence and determinants of psychiatric morbidity and the phenomenology of itch in PN compared to chronic urticaria and vitiligo, which are typically itchy and non-itchy chronic dermatoses, respectively, but both with well-known psychological morbidity and impairment in QoL. Significant psychiatric morbidity was found in PN, comparable to that seen in the other two groups, being depressive and anxiety disorders being the most common individual diagnoses [4]. Furthermore, more males than females were found to be suffering from psychiatric comorbidities, which is contrary to most studies that report preponderance of females, likely due to biological and psychosocial factors [9]. A possible explanation is that males had an overall greater impairment in QoL, especially in the group of patients with PN [4].

However, all of those studies focused on outpatients with psychiatric disorders only, the psychiatric diagnoses were often self-reported, and the possible impact that treatment modalities (including the type and daily dosage of psychoactive drugs) may have not been considered. In this report, we aimed to retrospectively evaluate the occurrence and type of cognitive impairment in a series of inpatients with PN.

## 2. Methods

This case series was a retrospective chart review carried out at the Oasi Research Institute-IRCCS of Troina, Italy (Department of Neurology IC and Unit of Dermatology, Regional Reference Centre for Prevention, Diagnosis, and Treatment of Genodermatosis). All the patients with PN admitted to a referral neurological institute from September 2018 to March 2021 were screened.

The inclusion criteria were as follows: adults (>18 years) and a diagnosis of PN confirmed by a trained dermatologist (C.S.) with expertise in PN using standard clinical and laboratory diagnostic criteria [1]. The diagnostic workup of cognitive impairment and psychiatric comorbidities (including neuropsychological evaluation, brain imaging, and psychoactive medications taken) was carried out according to the latest diagnostic criteria by a trained neurologist (G.L.) expert in cognitive disorders. Patients with systemic diseases affecting the central nervous system (CNS) and/or peripheral nervous system (PNS), any other dermatological disease different from PN, or past or current substance or drug abuse were excluded. Treatment of PN included both local and systemic treatment as usual [3].

The work was performed in accordance with the Declaration of Helsinki of 1964 and its later amendments. Written informed consent was obtained from all the subjects involved in the study. Ethical review and approval were waived due to the nature of the report itself, which was a retrospective chart review based on clinical examinations only and within the routine diagnostic workup expected for these patients.

## 3. Results

**Figure 1:**
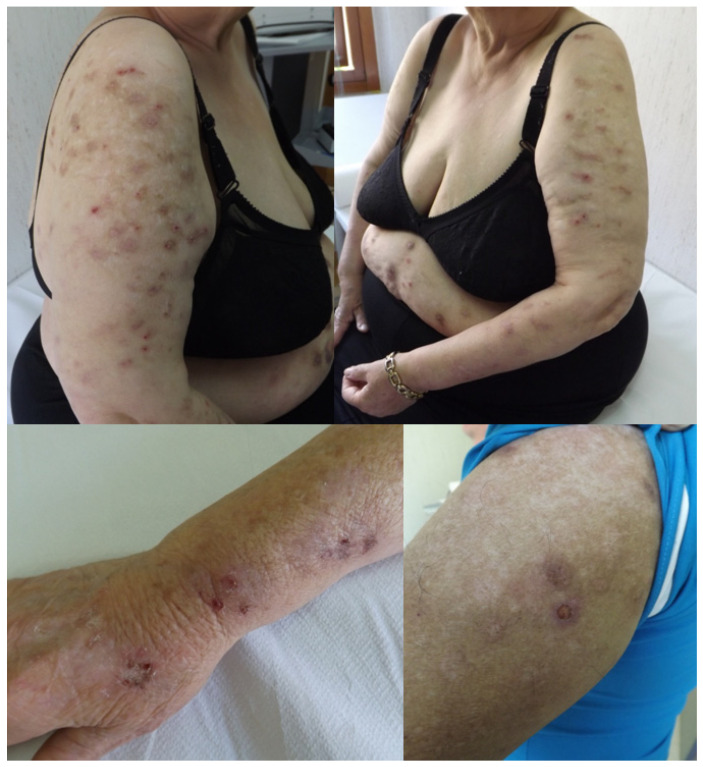
Examples of *prurigo nodularis* typically observed in two patients included in this report.

A total of 16 patients with PN (median age: 70 years, range 59.8–77.3 years; two males) were selected according to the inclusion/exclusion criteria from a total of 1806 hospital admissions diagnosed using neurocognitive assessment and neuroimaging. All the patients were Caucasian and of Sicilian ancestry. Figure 1 shows typical PN lesions observed in two patients, whereas Table 1 contains all the demographic features, neurological diagnosis, and psychiatric comorbidity as well as the psychoactive drugs taken by each participant. The sex distribution was significantly different from an expected 1/1 male/female ratio (chi-squared test, 5.24; *p* = 0.022).

Most of the patients had a neurodegenerative cognitive disorder, ranging from mild cognitive impairment (MCI) found in eight subjects to Alzheimer’s disease (AD) found in one, followed by mixed cognitive impairment (i.e., of both types, neurodegenerative and vascular) in six and multi-infarct vascular dementia (VaD) in one subject.

Regarding psychiatric manifestations, comorbid anxiety and depression were found in four patients, only depressive disorder—in four, only anxiety—in three, whereas bipolar disorder was diagnosed in two, and behavioral disturbances—in one patient with dementia; two patients had no psychiatric disease. Most of the patients were on combined treatment with benzodiazepines (of different types) and antidepressants (belonging to different classes: tricyclic antidepressants, selective serotonin reuptake inhibitors, serotonin–norepinephrine reuptake inhibitors, and trazodone), whereas the others were treated with different drugs (pregabalin for anxiety disorder; lithium for bipolar disorder; levetiracetam for focal epilepsy; promazine for dementia-related behavioral disturbances).

## 4. Discussion

### 4.1. Main Findings

To the best of our knowledge, this is the first report of cognitively impaired inpatients with PN and psychiatric comorbidities on psychoactive medications. First, we confirmed the association between psychiatric disorders, mostly depressive and anxiety disorders (often comorbid), followed by bipolar disorder and PN. However, cognitive impairment of both neurodegenerative (i.e., MCI/AD) and vascular origin (VaD/mixed dementia) may also be observed in these patients. Therefore, knowledge of these comorbid conditions can help guide the diagnostic workup and management of patients with PN. Finally, we confirmed the gender differences in chronic pruritus, with women being more commonly affected by PN and psychiatric symptoms.

These results build upon the findings of previous studies. A cross-sectional study of 909 patients found that PN was associated with concomitant depression more frequently than other dermatologic disorders, such as atopic dermatitis or psoriasis [10]. In a study of 109 dermatology inpatients experiencing chronic pruritus, more than 70% had some degree of psychiatric comorbidity, almost all developing after the onset of pruritus, with the severity of depression correlating with itch intensity [11]. Another study found a significant association of PN with anxiety, depression, and use of anxiolytics and antidepressants [8], thus confirming the results of this report. Finally, a recent study [12] found that PN was associated, with high odds, with virtually all mental health disorders, as well as with primary admission for psychiatric disorders, prolonged hospitalizations, and increased cost of hospitalization. The prolonged length of hospital stay observed in PN patients may reflect a more severe psychiatric disorder and/or additional days required to obtain consultation by a dermatologist in some inpatient settings [12].

Almost all our patients were taking psychoactive drugs; that might be important; however, in a large series of patients with chronic pruritus in the absence of skin disease, pruritus was found to be iatrogenic in only 7.1% of cases, none of whom was taking benzodiazepine or antidepressants [13]. On the other hand, antidepressants, such as duloxetine, paroxetine, and fluvoxamine, are also used for the treatment of PN [14].

### 4.2. Proposed Pathomechanisms

The mechanisms of association between PN and mental disorders are still unknown, being likely in a bidirectional relationship. Mental health disorders may result from shared mechanisms with PN, such as the upregulation of the IL-31 pathway implicated in the pathogenesis of PN [15]. A study of 218 patients with schizophrenia found that different single-nucleotide polymorphisms of IL-31 receptor A were associated with schizophrenia [16]. It is possible that the IL-31 pathway also plays a role in the pathogenesis of other psychiatric disorders, although little is known yet about its effects on cognitive impairment and symptoms of dementia. In addition, the increased psychiatric comorbidities may be attributable to PN, it being a highly symptomatic and debilitating chronic disorder; similar associations were found in other chronic inflammatory skin diseases [17,18,19,20,21,22,23]. Conversely, as already known, preexisting mental disorders may exacerbate both itch and the itch–scratch cycle in PN [12].

In addition to dermatoses, systemic conditions, infections, and psychiatric disorders, neurological diseases are also known to trigger PN and the frequently treatment-refractory itch–scratch cycle [24]. However, only PNS disorders causing compression and damage of peripheral nerves or a small-fiber neuropathy have been associated with PN [25], whereas, to date, no study has investigated effects on the CNS (such as cognitive impairment and dementia) in PN. In this context, there is evidence of increased cerebrovascular disease in PN, a finding which is in line with the results of this report. Compared with age- and sex-matched control subjects, patients with PN had approximately twice as high odds of cerebrovascular events, according to an epidemiologic study [26]. Moreover, this risk was also increased compared with patients with psoriasis after adjusting for age and sex. This is notable because patients with psoriasis are known to have an increased risk of atherosclerotic conditions (including cerebrovascular disease) and increased mortality compared with the general population [27].

Recently, it has been emphasized that CNS or PNS disorders (such as multiple sclerosis, brain tumors or abscesses, cerebral or spinal infarcts, brachioradial pruritus, notalgia paresthetica, postherpetic neuralgia, small-fiber neuropathy) can lead to neuropathic itching [28], as also observed in other dermatological diseases [29,30,31]. In particular, it is known that neuropathic itching is accompanied by other sensory symptoms, such as paresthesia, hyperesthesia, and hypoesthesia, and that patients with PN often complain of these symptoms [24]. Overall, however, data demonstrating a relationship between PN and neurological disorders are still insufficient. One retrospective study only [32] on 80 females with PN categorized according to the accompanying diseases (dermatological, systemic, psychogenic, neurological, mixed, or of undetermined origin) found a neurological disease in 14 (17.5%) of them, peripheral neuropathy (four patients) and migraine (four patients) being the most frequent diagnoses. Of note, one patient had AD and one had a cerebrovascular accident; although the authors acknowledged that the coexistence of these conditions could be merely coincidental, they cannot exclude their role as cofactors contributing to the worsening or chronicity of the symptoms [24,33].

### 4.3. Limitations

Limitations of this study are the small sample size, retrospective design, non-blinded nature of the assessments (which makes the results less objective), and lack of a control or non-PN group. In particular, without a reference population without any dermatological disease, the actual prevalence of comorbid cognitive impairment and psychiatric disorders cannot be measured.

Another caveat is that determining the temporal relationship between PN and the development of psychiatric disorders cannot be established. Indeed, in the analysis of this relationship, a “post hoc fallacy” is possible. In other words, PN may increase the risk of developing mental disorders, but preexisting mental disorders may also increase the risk of PN [12]. Given the paucity of data confirming these associations, more research is needed to quantify the full burden of neurological and psychiatric comorbidity in PN. In particular, whether these conditions contribute to the pathogenesis of PN or are exacerbated by chronic intense refractory pruritus deserves further study [6]. 

Lastly, the occurrence of PN observed in a sample of inpatients admitted to a referral neurological institute may not reflect the actual prevalence in the general population. 

Taken together, these limitations prevent an in-depth assessment of the results, which only provide a descriptive analysis of the observed findings.

## 5. Conclusions

Cognitive impairment can be observed in PN. In addition to screening for psychiatric diseases and initiating appropriate treatment or referral, clinicians may also consider the presence of cognitive impairment in PN of both neurodegenerative and vascular origin. Studies in controlled samples are needed to determine the mechanisms for mental health disorders in PN and to develop evidence-based prevention and treatment strategies for the neuropsychiatric involvement in PN.

## Figures and Tables

**Table 1:** Main characteristics of the patients with *prurigo nodularis* included in this report.

No.	Age (Years)	Sex	Neurological Diagnosis at Discharge	Psychiatric Comorbidity	CNS Drug Taken (Daily Dosage)
1	69	F	Mild AD, SIVD	-	-
2	73	F	MCI, diffuse cortical–subcortical atrophy	Anxiety and depressive disorder	Prazepam 10 mg Escitalopram 10 mg
3	61	F	MCI, frontal lobe atrophy	Anxiety and depressive disorder	Oxazepam 15 mg Venlafaxine 75 mg
4	79	F	MCI, cortical atrophy	-	-
5	67	F	MCI, SIVD	Depressive disorder	Paroxetine 20 mg
6	78	F	Mild AD, SIVD	Anxiety disorder	Pregabalin 75 mg
7	79	F	MCI, cortical–subcortical atrophy	Depressive disorder	Sertraline 50 mg
8	53	F	MCI, cortical–subcortical atrophy	Bipolar disorder	Duloxetine 60 mg Lithium 300 mg (×3) Trazodone 75 mg
9	56	F	MCI, posterior cortical atrophy, focal epilepsy	Anxiety and depressive disorder	Alprazolam 1 mg Paroxetine 20 mg Levetiracetam 500 mg (×2)
10	81	F	MCI, parietal lobe atrophy, SIVD	Bipolar disorder	Paroxetine 20 mg Nortriptyline 25 mg
11	55	M	MCI, SIVD	Anxiety and depressive disorder	Escitalopram 10 mg
12	71	F	Moderate multi-infarct vascular dementia	Behavioral disturbances in dementia	Promazine 10 gtt (×3)
13	55	M	MCI, cortical–subcortical atrophy	Depressive disorder	Alprazolam 0.25 mg (×2) Venlafaxine 75 mg
14	77	F	Moderate AD	Anxiety disorder	Medazepam 4 mg
15	76	F	MCI, mild diffuse cortical atrophy	Depressive disorder	Escitalopram 10 mg
16	69	F	MCI, SIVD	Anxiety disorder	Delorazepam 1 mg

Abbreviations: AD: Alzheimer’s disease; CNS: central nervous system; F: female; M: male; MCI: mild cognitive impairment; SIVD: subcortical ischemic vascular disease.

## Data Availability

Data is contained within the article.
